# Day Biting Habits of Mosquitoes Associated with Mangrove Forests in Kedah, Malaysia

**DOI:** 10.3390/tropicalmed3030077

**Published:** 2018-07-23

**Authors:** Tengku Nur Saffawati T. Ismail, Nur Faeza A. Kassim, Azimah A. Rahman, Khairun Yahya, Cameron E. Webb

**Affiliations:** 1 School of Biological Sciences, Universiti Sains Malaysia, Penang 11800, Malaysia; saffawati.ismail@gmail.com (T.N.S.T.I); khairun@usm.my (K.Y.); 2 School of Humanities, Universiti Sains Malaysia, Penang 11800, Malaysia; azimahrahman@usm.my; 3 Medical Entomology, NSW Health Pathology, Level 3 ICPMR, Westmead Hospital, Westmead 2145, Australia; cameron.webb@health.nsw.gov.au; 4 Marie Bashir Institute of Infectious Diseases and Biosecurity, University of Sydney, Sydney 2000, Australia

**Keywords:** mangrove, forest, *Verrallina butleri*, *Aedes albopictus*, *Aedes*, *Culex*, biting habits

## Abstract

Due to conservation and rehabilitation efforts, mangrove forests represent some of the largest environmental niches in Malaysia. However, there is little information on the potential risks posed by mosquitoes that are directly and indirectly associated with mangrove forests. To study the potential health risk to humans active within and in close vicinity of mangrove forests, this research focused on the day biting habits of mosquitoes in mangrove forests of Kedah, Malaysia. The bare leg catch (BLC) method was used to collect adult mosquitoes during a 12-h period from 7:30 a.m. to 7:30 p.m. in both disturbed and less disturbed areas of mangroves. In total, 795 adult mosquitoes from 5 genera and 8 species were collected, and over 65% of the total mosquitoes were collected from the less disturbed area. The predominant species from the less disturbed area was *Verrallina butleri*; in the disturbed area the dominant species was *Culex sitiens*. The peak biting hour differed for each species, with *Aedes albopictus* and *Cx. sitiens* recorded as having a bimodal biting activity peak during dawn and dusk. For *Ve. butleri* an erratic pattern of biting activity was recorded in the less disturbed area but it peaked during the early daytime for both collection points. Overall, the distinct pattern of day biting habits of mosquitoes within mangroves peaked during dawn and dusk for the less disturbed area but was irregular for the disturbed area throughout the day. The presence of vectors of pathogens such as *Ae. albopictus* for both areas raises the need for authorities to consider management of mosquitoes in mangrove forests.

## 1. Introduction

Mosquito-borne disease is a major concern in Southeast Asia, especially in Malaysia where outbreaks of dengue have a significant impact on human health. The mosquitoes typically associated with these outbreaks are *Aedes aegypti* and *Aedes albopictus*, which are most commonly associated with artificial water-holding containers found in urban environments. Greater interest must to paid to other environments and the mosquitoes associated with them to better understand and manage mosquito-borne disease outbreak risk. Mangrove forests are extensive habitats found in the intertidal zone between land and sea, forming a buffer against storm surge and tidal waves [1]. The biological and ecological importance of these habitats has not generally been appreciated and human activity has caused substantial damage and disruption, with losses of one quarter of total mangrove areas in the last 20 years [2]. While some aspects of mangrove wildlife have been studied, there is a paucity of information available on mosquitoes found in these habitats in Malaysia. Mosquitoes are a diverse group of insects that are present across a wide range of environmental niches in both tropical and temperate regions, and while they may play important ecological roles, they also represent potentially significant pest and public health risks [3].

Mangroves provide unique ecological niches and permit diverse fauna to adapt to these environments [4]. In mangrove forests in Southeast Asia the presence of mosquitoes is acknowledged as a component of the mangrove ecosystem [5]. There is a suite of mosquitoes recorded across tropical regions that may utilize habitats within mangrove forests that provide potentially diverse habitats for mosquitoes. Notwithstanding the ground pools that form following rainfall or tidal inundation, tree holes in the mature trees may fill with water following rainfall and accumulation of rubbish, particularly plastic containers and other debris, may provide habitats for container-inhabiting mosquitoes otherwise typically associated with urban habitats.

Previous research has recorded the presence of a few vectors of mosquito-borne pathogens in mangrove forests such as *Ae. albopictus*, *Ae. aegypti*, and *Culex quinquefasciatus* [6], although these mosquitoes may not be directly associated with estuarine ground pools in these habitats. The potential risk associated with mosquito-borne disease is a concern, with *Ae. albopictus* being a vector of dengue viruses (DENV) and *Cx. quinquefasciatus* being a vector of the West Nile virus (WNV) [7]. Another mosquito, typically associated with saline-brackish ground pools mangrove forests, is *Culex sitiens*, a potential vector of the Japanese encephalitis virus (JEV) [8]. A positive case was recorded in Selangor in 1992 [9].

Understanding the ecological behavior of mosquitoes in mangrove forests is critical given the risk of mosquito-borne diseases. Dengue is endemic in Malaysia but little is known how the mosquito populations associated with mangroves may drive increased public health risks and how these risks should be managed by local authorities. This is especially important given the increased focus on mangrove conservation across Southeast Asia to minimize the impacts of sea level rise and increasingly frequent extreme weather events, and to provide protection to local wildlife. The biting behavior of mosquitoes is a conventional way to study mosquito activity and to define possible relationships between mosquito activity and outbreaks of mosquito-borne diseases. Due to the nocturnal behavior of mosquitoes, most research on biting activity has been conducted between sunset and sunrise. However, a study conducted by Chen et al. [10] on the biting activity of *Ae. albopictus* in urban areas reported the highest biting peak as occurring between 6:00 and 9:00 a.m. and between 3:00 and 8:00 p.m. Mangrove forests represent an important refuge habitat for adult mosquitoes due to high humidity and shaded canopy [11]. Previous research by Haddow [12] showed that the biting pattern of *Aedes* mosquitoes responding to bare leg catch sampling techniques over a 24-h period peaked for an hour at dawn and dusk.

The aim of this research project was to document the daytime activity, especially biting habits, of mosquitoes associated with mangrove forest and provide recommendations on future management of associated public health risks.

## 2. Materials and Methods

### 2.1. Study Area

**Figure 1:**
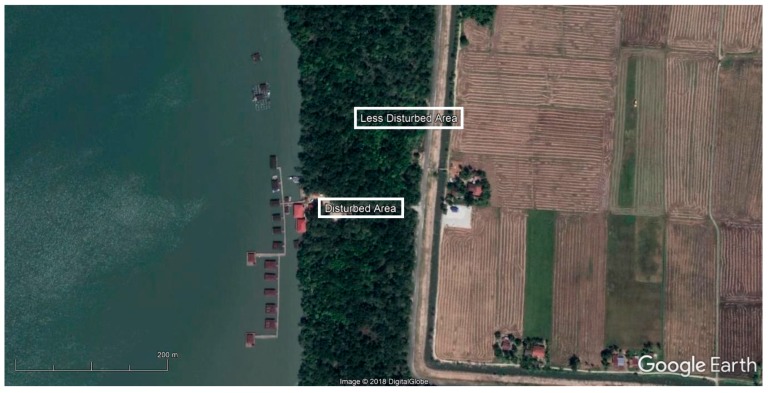
Segantang Garam, Merbok, Kedah mangrove forest.

The experiments were conducted at Segantang Garam, Merbok, Kedah mangrove forest (Figure 1). Merbok mangrove forest is situated between (05°38′52.8″ N, 100°24′30.5″ E) and (05°38′54.8″ N, 100°24′30.5″ E), and is located 9 km from Sungai Petani, Kedah. The study was performed in the inland zone, which consists of mostly *Rhizophora* mangrove species. Mosquito collections were performed in two different areas: disturbed and less disturbed areas.

The characterization of the collection areas was made according to the distance from the shoreline, the presence of artificial containers, and mangrove canopy cover. The disturbed area is located at latitude 05°38.790′ N and longitude 100°24.471′ E, and is situated between the mangrove-lined shoreline and agricultural areas. The less disturbed area is located at latitude 05°38.855′ N and longitude 100°24.506′ E, which is further away from the shoreline and residential areas. The canopy cover in the disturbed area is also very light compared to the heavy and crowded canopy in the less disturbed area.

### 2.2. Mosquito Collection Procedure

From March to June 2015, three replicates at 12 time points using the daytime bare leg catch (BLC) method were performed to collect adult mosquitoes [13]. Sampling was conducted at two different collection sites. At each collection point, a total of six volunteers collected mosquitoes, with each volunteer sitting on a wooden stool with rolled up pants to expose a bare leg. The procedure started at 7:30 a.m. and ended at 7:30 p.m. For each one hour of BLC sampling, 45 min was allotted to collecting samples with 15 min of rest. When the mosquitoes landed, they were collected by using an aspirator and stored in a test tube. The samples then returned to the laboratory and stored at 0 °C prior to identification. Prior to the experiment, all volunteers were provided with information on the potential risks of the experiments for written informed consent and were also advised about personal protection measures before and after experiments. Volunteers were also monitored following the experiment for any symptoms of mosquito-borne disease.

### 2.3. Identification

The samples were then identified according to morphological characteristics of mosquito-based identification keys [14,15]. The samples were identified and the abundance of each species was recorded according to the time point of collection.

### 2.4. Climatic Data

During the duration of sampling at study sites, meteorological parameters such as temperature (°C), humidity (%), and light intensity (kLux) using a hygro-thermometer and handheld lux-meter were recorded in both disturbed and less disturbed areas each hour.

### 2.5. Data Analysis

All mosquitoes species collected at each time points were pooled to determine the overall biting pattern throughout the day. The Shannon–Weiner Index was used to calculate the diversity index of mosquitoes in disturbed and less disturbed areas. The mean number of mosquitoes collected in disturbed and less disturbed areas were compared by ANOVA with the following factors (time and species) after log + 1 normalization (Shapiro–Wilk test). The paired t-test was used for mean difference between disturbed and less disturbed areas. The statistical analysis was considered significant when *p*-value was <0.05.

## 3. Results

### 3.1. Species Composition of Mangrove Mosquitoes in the Merbok Mangrove Forest

There was little difference in the mean relative humidity (RH) or mean temperature (MT) between the disturbed (RH = 76.3% and MT = 31.5) and less disturbed (RH = 80.0% and MT = 30.3 °C) areas. As shown in Table 1, in total 795 mosquitoes were collected in the study sites of disturbed and less disturbed area of Merbok mangrove forests. These mosquitoes belonged to five genera and eight species, with seven species recorded from the disturbed area compared to only six species from the less disturbed area. Overall, there was a substantially greater number of mosquito specimens collected in the less disturbed areas (534 mosquitoes) compared to the disturbed areas (261 mosquitoes).

The three most abundant mosquitoes across the two areas were *Ae. albopictus*, *Ve. butleri*, and *Cx. sitiens*. Both *Ve. butleri* and *Cx. sitiens* were more abundant in less disturbed areas, while there was relatively little difference in the abundance of *Ae. albopictus* between the two areas.

Based on species composition of mangrove mosquitoes, the Shannon–Weiner diversity index was constructed, and the results show 1351 for disturbed areas and 1078 for less disturbed areas. The diversity index shows a higher diversity for the disturbed area compared to the less disturbed area despite the overall lower abundance of mosquitoes in the disturbed area. The paired *t*-test on the mean difference between disturbed and less disturbed areas shows there was a significant difference between the two mean mosquito populations (*t* = −2.218, *p* < 0.05).

### 3.2. Day Biting Habits of Mosquitoes in Merbok Mangrove Forests

There were distinct differences in the peak periods of activity of individual mosquito species in both the disturbed and less disturbed areas in the Merbok mangrove forests, especially for the predominant species collected in the mangroves, *Cx. sitiens* and *Ve. butleri*. For *Cx. sitiens* the peak biting activity was recorded from 6:30 to 7:30 p.m. in the disturbed and less disturbed areas, while for *Ve. butleri* the highest biting activity was recorded from 6:00 to 7:30 p.m. in the disturbed area and from 7:30 to 8:30 a.m. in the less disturbed area.

For *Ae. albopictus* constant biting activity was recorded throughout the day, but it peaked at dawn and dusk (from 7:30 a.m. to 8:30 a.m. and 6:30 p.m. to 7:30 p.m., respectively) in disturbed areas. However, biting activity for this species peaked at dawn (7:30 to 8:30 a.m.) and dropped throughout the day in less disturbed areas. Statistical ANOVA demonstrated a significant difference according to species for disturbed areas (*F* = 14.973, *p* < 0.05) and less disturbed areas (*F* = 26.210, *p* < 0.05). The same statistical analysis test for collection points (hour) also showed a significant difference for disturbed areas (*F* = 50.139, *p* < 0.05) and less disturbed areas (*F* = 64.512, *p* < 0.05).

## 4. Discussion

This study is one of the first to document day biting mosquito activity within the Merbok mangrove forests. This information on biting habits of mosquitoes active during the day in the mangrove forests, especially vector species, is another step forward in assessing the local risks of mosquito-borne disease and, given that this study demonstrates that biting mosquitoes are active during the day in mangrove forests, efforts must be made to minimize the interactions of the potential vectors with humans. This requires local authorities to consider mosquito management during periods of mosquito-borne disease outbreak and where mangrove conservation is undertaken as there may be potential for increased mosquito activity.

This study confirmed the presence of mosquitoes that may pose a risk of transmitting pathogens within the mangroves. *Aedes albopictus* is the species of greatest concern given its role in the transmission of dengue, chikungunya, and Zika viruses. However, other mosquitoes such as *Ve. butleri* and *Cx. sitiens* have also been implicated as vectors of pathogens including JEV. Further work is required to determine the role of mosquitoes associated with mangrove habitats in the transmission of other zoonotic pathogens, especially in situations where degradation of habitats enhances mosquito productivity or changes in vertebrate reservoir hosts of pathogens (e.g., birds, mammals) occur. Testing the collected mosquito specimens for the presence of pathogens was beyond the scope of this study but should be considered in future. It is critical to understand the prevalence of mosquito-borne pathogens to better manage potential health risks and, as a consequence, local authorities may need to consider routine surveillance programs where potential risks have been identified.

With regard to managing habitats in mangrove forests, it is important to note that not all mosquitoes detected as adults in human landing collections are directly associated with larval habitats within the mangroves. Typically, natural ground pool habitats are estuarine in nature and species such as *Ve. butleri* and *Cx. sitiens* will be directly associated with these tidally-influenced environments. However, species such as *Ae. albopictus* are likely to either be associated with rain-filled tree hole habitats or water-holding containers (e.g., accumulated plastic containers, glass bottles, etc.) within the mangroves or similar habitats adjacent to the mangroves, where adult mosquitoes disperse into the area seeking refuge and/or blood meals. This is an important consideration when managing human waste around mangrove forests, where dumping of plastic containers and other items that may hold water could enhance local conditions for the production of mosquitoes.

It was expected that for the disturbed area (which is closer to residential or human dwelling areas with the presence of artificial containers as breeding sites of mosquitoes), a greater abundance of container-inhabiting mosquitoes might be active. Meanwhile, for the less disturbed area deep in the mangrove forest, it was expected that there would likely be fewer artificial containers present. The results of this investigation confirm the presence of a major dengue vector, *Ae. albopictus*, across both disturbed and less disturbed areas. The result highlights the need to consider the potential mosquito populations associated with what may be considered undisturbed mangrove habitats as being as potentially important as highly disturbed habitats for mosquito production. A detailed observation during the experiment has shown positive results with respect to the presence of mosquito larvae in natural habitats such as tree holes, and also a great abundance of mosquito larvae in artificial containers such as discarded tires. It is noteworthy that studies from Australia investigating mosquito populations of mangroves in urban areas demonstrated that container-inhabiting mosquitoes, such as *Aedes notoscriptus*, are abundant in mangrove areas adjacent to heavily urbanized land use [16] suggesting that the abundance of potential mosquito habitats may be influenced by the surrounding environments. This may extend beyond the accumulation of rubbish and other debris to degradation of mangroves and increased availability of tree holes.

In this study, the predominant species of the less disturbed area was *Ve. butleri*, for which biting peaked from 6:30 to 7:30 p.m. in disturbed areas and from 7:30 to 8:30 a.m. in less disturbed areas. The results are similar to a study case in Taman Alam, Selangor, with respect to areas previously made up of mangrove areas that were developed into residential areas [17]. This species is predominantly found in brackish water habitats and is closely associated with ground pools in swampy or brackish water zones [18]. Further investigation is required to better understand the habitat associations with this species, as it may be critical for mosquito management in areas where mangrove habitats are being created or rehabilitated. Understanding the role of tidal cycles and rainfall in driving mosquito abundance will greatly assist mosquito management.

In disturbed area, *Cx. sitiens* is the most dominant species and recorded biting activity peaked at 6:30 to 7:30 p.m. The results complement a previous study in Thailand, which showed that *Cx. sitiens* was the most dominant species found in association with brackish water habitats created following inundation by a tsunami [19]. Previous research conducted in Papua New Guinea revealed that *Cx. sitiens* is a nocturnal species, with relatively no adult mosquitoes collected during the day [20]. However, many species are often found biting during the day in shaded habitats when abundant and it is interesting to note that disturbed mangrove habitats may be more likely to contain ground pools following rain and tidal inundation, so the disturbed nature of these habitats may produce a greater abundances of mosquitoes. Other than estuarine and brackish water ground pools, this species is also commonly found in nipah palm, salt marsh, and water-holding containers such as cans, bottles, and cement tanks [21].

*Aedes albopictus* was recorded as the second most abundant human biting species in disturbed areas, with a peak biting hour from 6:30 to 7:30 p.m. However, the result also shows a distinct number of *Ae. albopictus* collected from 7:30 to 8:30 a.m. and throughout morning hours, supporting previous studies that found their biting behavior peaked during dawn and dusk in a 24-h cycle [10,22]. It is generally considered that *Ae. albopictus* is an urban pest that has a preference for biting humans. However, unlike the more significant DENV vector *Ae. aegypti*, which is generally almost exclusively associated with artificial water-holding containers, *Ae. albopictus* is also found in sylvatic habitats. Our results showed a relatively high number of mosquitoes collected in mangrove forests, providing strong evidence that *Ae. albopictus* is a potential public health problem in both suburban and adjacent non-urban environments [23]. While human activity may be lower within extensive mangrove-dominated environments, the mosquito’s tendency to feed on other mammals, reptiles, and avian species [24,25] will facilitate the sustainment of mosquito populations in these environments. Our results should not be considered indicative that populations of *Ae. aegypti* cannot exist in mangrove forests; without further study of the role of natural and artificial water-holding containers it is difficult to reliably conclude future risk associated with specific habitats within these areas.

This study confirms that there are health risks posed by mosquitoes associated with mangroves. Further research is required to understand the habitat associations of mosquitoes found within these environments. In particular, key pest and vector species such as *Ae. albopictus* are directly associated with tree-hole environments within the mangroves or rubbish and other debris washed into the mangroves by tides. Similarly, determining the habitat preferences of the most common mosquitoes, *Ve. butleri* and *Cx. sitiens*, within the mangroves of Malaysia will greatly assist the understanding of risks posed by other mosquito-borne pathogens. The results of such studies may make a valuable contribution to the design of mangrove management strategies, especially where accumulation of plastic rubbish and debris may be found to be providing mosquito habitat. Local authorities may need to plan specific actions targeting clean-up efforts of this waste. As a consequence, notwithstanding the potential environmental impacts on mangroves, pollution and degradation may have indirect human health impacts should mosquito populations become more abundant.

## 5. Conclusions

This study confirmed that pest and vector mosquitoes were present throughout both disturbed and less disturbed areas of Merbok mangroves. The biting activity of mosquitoes in mangrove forests mimics the biting habits of mosquitoes in urban and pre-urban areas, which peaked at dawn and dusk, but shows greater potential for biting activity throughout the day, especially in disturbed areas. The urbanization and human mobility near the mangrove areas has caused the expansion of disturbed areas and the addition of more sources for mosquito vector habitats. Appropriate measures such as the elimination of artificial debris that acts as mosquito breeding sites should be taken to ensure mangrove areas do not provide habitats for these mosquito vectors.

## Figures and Tables

**Table 1:** Number of individuals per each mosquito species collected in disturbed and less disturbed areas of Merbok mangrove forest.

Species	Disturbed Areas	Less Disturbed Areas
*Verrallina butleri*	74	299
*Culex sitiens*	89	153
*Aedes albopictus*	79	61
*Armigeres subalbatus*	8	18
*Culex tritaeniorhynchus*	9	2
*Mansonia uniformis*	1	0
*Aedes vigilax*	1	0
*Aedes vexans*	0	1
Total	261	534
